# Acquisition of EMT phenotype in the gefitinib-resistant cells of a head and neck squamous cell carcinoma cell line through Akt/GSK-3*β*/snail signalling pathway

**DOI:** 10.1038/bjc.2012.24

**Published:** 2012-02-07

**Authors:** S Maseki, K Ijichi, H Tanaka, M Fujii, Y Hasegawa, T Ogawa, S Murakami, E Kondo, H Nakanishi

**Affiliations:** 1 Division of Oncological Pathology, Aichi Cancer Center Research Institute, 1-1 Kanokoden, Chikusa-ku, Nagoya 464-8681, Japan; 2 Department of Otolaryngology-Head & Neck Surgery, Nagoya City University Graduate School of Medical Sciences, 1 Kawasumi, Mizuho-cho, Mizuho-ku, Nagoya, Japan; 3 Division of Molecular Oncology, Aichi Cancer Center Research Institute, 1-1 Kanokoden, Chikusa-ku, Nagoya, Japan; 4 Department of Head and Neck Surgery, Aichi Cancer Center Hospital, 1-1 Kanokoden, Chikusa-ku, Nagoya, Japan; 5 Department of Otolaryngology, Aichi Medical University School of Medicine, Nagakute-cho, Aichi, Japan

**Keywords:** EMT, gefitinib, EGFR, Akt, GSK-3*β*, snail

## Abstract

**Background:**

Epithelial mesenchymal transition (EMT) is known to be associated with chemoresistance as well as increased invasion/metastasis. However, the relationship between EMT and resistance to an epidermal growth factor receptor (EGFR) -targeting drug in head and neck squamous cell carcinoma (HNSCC) remains unknown. In this study, we investigated the acquisition of EMT by gefitinib in HNSCC cell line (UMSCC81B).

**Methods:**

We isolated fibroblastoid variant (81B-Fb) from gefitinib-resistant UMSCC81B-GR3 cells obtained after increasing the doses of gefitinib treatment *in vitro* and examined EMT and its underlying mechanism.

**Result:**

81B-Fb cells exhibited fibroblast-like morphology, increased motility, loss of E-cadherin, acquisition of vimentin and snail expression. In 81B-Fb cells, downregulation of EGFR, which is mediated by increased ubiquitination, and activation of downstream protein kinase B (Akt), glycogen synthase kinase-beta (GSK-3*β*) signalling and upregulation of snail expression were observed compared with UMSCC81B cells. LY294002, but not U0126, suppressed foetal bovine serum or heregulin-*β*1-induced phosphorylation of Akt/GSK-3*β* and snail expression together with the inhibition of 81B-Fb cell motility. Furthermore, forced expression of EGFR resulted in partial restoration of gefitinib sensitivity and reversal of EMT.

**Conclusion:**

These results suggest that EMT in the gefitinib-resistant cells is mediated by the downregulation of EGFR and compensatory activation of Akt/GSK-3*β*/snail pathway.

Epidermal growth factor receptor (EGFR) has been implicated in the pathogenesis of head and neck squamous cell carcinoma (HNSCC). Epidermal growth factor receptor is overexpressed in up to 90% of HNSCC (Kalyankrishna and Grandis, 2006). Targeting EGFR using small molecule EGFR-tyrosine kinase inhibitors (TKI) such as gefitinib or monoclonal antibodies against EGFR (cetuximab) abrogates tumour growth in the preclinical HNSCC models (Erjala *et al*, 2006; Nozawa *et al*, 2006).

Cetuximab, a chimeric, mouse-human IgG1 monoclonal antibody, was approved by the Food and Drug Administration (FDA) at 2006 in combination with radiation for the treatment of HNSCC. More recently, cetuximab plus chemotherapy was shown to be effective in the recurrent/metastatic HNSCC (Vermorken *et al*, 2008). On the other hand, gefitinib, an orally active, quinazoline TKI, has not been approved in HNSCC unlike non-small-cell lung carcinoma (NSCLC) cases. In HNSCC, a phase II clinical study examining the combination of radiotherapy and gefitinib reported 32% complete remission and 53% partial remission, while a phase III clinical trial of gefitinib in combination with methotrexate for the treatment of recurrent/metastatic HNSCC could not demonstrate a significant survival advantage (Stewart *et al*, 2009). Therefore, the clinical benefit of gefitinib in combination with chemotherapy or radiotherapy is still inconsistent. However, there is no doubt this agent is still one of the potential EGFR-targeting agents for HNSCC. As for gefitinib resistance, mutation such as T790M is well known to confer the resistant phenotype in NSCLC (Chen *et al*, 2009). However, such EGFR mutation is uncommon in patients with HNSCC (Hama *et al*, 2009) and truncation mutation EGFR variant III (EGFRvIII), which is relatively prevalent in HNSCC does not correlate with resistance to EGFR-TKIs (Sok *et al*, 2006). Therefore, the mechanism of gefitinib resistance in HNSCC remains largely unknown.

Epithelial mesenchymal transition (EMT) is known to be deeply involved in cancer progression and metastasis. Epithelial mesenchymal transition is characterised by the loss of proteins involved in cell junctions such as E-cadherin, and the expression of mesenchymal markers such as vimentin (Klymkowsky and Savagner, 2009). Acquisition of EMT features has also been associated with chemoresistance acquired after standard chemotherapy (Iwatsuki *et al*, 2010). Furthermore, EMT is reportedly associated with the resistance to gefitinib and erlotinib in NSCLC (Frederick *et al*, 2007; Uramoto *et al*, 2010). These disregulated genes such as loss of E-cadherin and gain of vimentin have been identified as predictive marker for high-risk HNSCC tumours such as gefitinib resistance, but it remains unclear whether overexpression of EMT-associated genes are directly responsible for gefitinib resistance.

In the present study, to clarify these questions, we newly developed a cell line showing EMT by repeated gefitinib treatment using HNSCC cell lines. With this model, we found the link between EMT and gefitinib resistance in HNSCC cell line. The potential mechanism of the link between EMT and gefitinib resistance through EGFR downregulation will be discussed.

## Materials and methods

### Reagents

Gefitinib was purchased from Bio Australis (NSW, Australia). Human recombinant EGF was obtained from R&D Systems (Minneapolis, MN, USA). Phosphatidylinositol-3-kinase (PI3K) inhibitor (LY294002) and mitogen-activated protein/extracellular signal-regulated kinase kinase (MEK) 1/2 inhibitor (U0126) were obtained from Cell Signaling Technology (Beverly, MA, USA). MG132, a proteasome inhibitor, was obtained from Calbiochem (San Diego, CA, USA). Antibodies used were as follows: for western blotting analysis, mouse monoclonal antibodies to total EGFR (Thermo Scientific, Waltham, MA, USA), phospho-Erk1/2 (Thr202/Tyr204) (Cell Signaling Technology), E-cadherin (BD Transduction Laboratories, Rockville, MD, USA), vimentin (Dako, Glostrup, Denmark) and rabbit polyclonal antibodies to phospo-EGFR (Tyr845) (Abcam, Cambridge, UK), total Erk1/2, total Akt, phospho-Akt (Ser473), total GSK-3*β*, phospo-GSK-3*β* (Ser9), snail, twist (Cell Signaling Technology) and *β*-actin (Sigma Aldrich, St Louis, MO, USA) were used. For immunoprecipitation, mouse monoclonal antibody to EGFR (Thermo Scientific) and rabbit monoclonal antibody to ubiquitin (Dako) were used. For immunohistochemistry, mouse monoclonal antibody to E-cadherin (Dako) and vimentin (Dako) were used.

### Cell line and cell culture

The UMSCC81B cells (HNSCC cell line) were kindly donated by Dr Thomas E Carey, Laboratory of Head and Neck Cancer Biology at the University of Michigan. This cell line was maintained in Dulbecco's modified Eagle's Medium (DMEM) (Sigma Aldrich) supplemented with 10% foetal bovine serum (FBS) (Invitrogen, Carlsbad, CA, USA) in a humidified atmosphere of 5% CO_2_ at 37 °C.

### Isolation of gefitinib-resistant cell line

UMSCC81B cells cultured in DMEM with 10% FBS were continuously exposed to gefitinib at stepwisely increased concentrations of 20, 40 and 50 *μ*M each for 1 week. After each gefitinib exposure, remaining cells were cultured in gefitinib-free growth medium until stable growth was restored. After three gefitinib exposures, gefitinib-resistant cell line (UMSCC81B-GR3) was established in which a small number of variant cells with fibroblastic morphology appeared around epithelial cell nest. Pure fibroblastoid tumour cells were then isolated by mechanical scratching epithelial cells. Such fibroblastoid tumour cells were successfully cultured for more than half year without any morphological change, after which pure fibroblastoid tumour cell line designated 81B-Fb was established.

### Epidermal growth factor receptor transfection

Human EGFR expression vector, pLenti6/V5-wt EGFR with blasticidin-resistance gene was kindly provided by Dr M Sato (Nagoya University School of Medicine, Japan). Stable transfectants of 81B-Fb cells with EGFR plasmid were isolated after selection with blasticidin (Invitrogen) at 20 *μ*g ml^−1^, and further screened with quantitative RT–PCR analysis for EGFR expression. Stable clones with high EGFR expression, designated as Tf-1 and Tf-2 were used in this study. 81B-Fb cells transfected with control vector was used as a negative control.

### *In vitro* cell growth assay

Cells were harvested with trypsin/EDTA, plated at 1 × 10^4^ cells per 96-well plastic plate in DMEM with 10% FBS, and then treatment with increasing doses of gefitinib (0.1, 1 and 10 *μ*M) started on day 1. The number of viable cells was counted on day 4. In the cell growth assay without gefitinib treatment, the number of viable cells was counted on day 2, 4 and 6 in triplicate by haemocytometer.

### Wound closure assay

Cell migration was assessed by the ability of the cells to migrate into a cell-free area. Briefly, cells were plated 1 × 10^5^ cells in growth medium on 24-well plates and grown for 24 h to reach confluence. The monolayers were then wounded by scratching with a plastic yellow pipette tip. After washing, the cells were incubated in growth medium with or without any inhibitors for 8–12 h and observed under a microscope. The wound closure was estimated as the ratio of the remaining wound area relative to the initial wounded area. Experiments were repeated at least three times.

### Western blot analysis

The cultured cells grown on 6 cm dishes with any conditions were lysed at 4 °C in lysis buffer and whole-cell lysates were prepared by sonication and centrifugation as described previously (Yokoyama *et al*, 2006). Protein concentration was determined by Lowry assay (DC Protein Assay, Bio-Rad, Hercules, CA, USA), and 50 *μ*g cell aliquots were directly lysed in Laemmli sample buffer for subsequent immunoblotting with antibodies. Whole-cell lysates were separated by SDS–PAGE, and transferred to Immune-Blot PVDF membrane (Bio-Rad) and immunoblotted with antibodies. Bound antibodies were visualised using Super Signal West Pico (or Dura) chemiluminescence substrate (Thermo Scientific).

### Immunoprecipitation of EGFR

The cultured cells grown on 10 cm dishes were serum-starved for 24 h, pretreated with 10 mM MG132 for 2 h and then stimulated with 100 ng ml^−1^ EGF for 15 min. The cells were lysed with 500 *μ*l of lysis buffer (10 mM HEPES, pH 7.5, 200 mM NaCl, 30 mM Sodium pyrophosphate, 50 mM sodium fluoride, 1% Triton X-100 and phosphatase inhibitor cocktail), scraped, sonicated and centrifuged. The resultant supernatant was collected as whole-cell lysates and was used for immunoprecipitation. Fifty *μ*l of Dynabeads Protein G (Invitrogen, Oslo, Norway) and 2 *μ*g of EGFR antibody were mixed and incubated with rotation for 10 min at room temperature. The lysates were mixed with this Dynabeads-antibody complex in 500 *μ*l of TNT buffer (20 mM Tris-HCl, pH 7.5, 200 mM NaCl, 1% Triton X-100 and phosphatase inhibitor cocktail) and rotated for 10 min at room temperature. After washing, lysates were eluted with elution buffer, separated by SDS–PAGE and immunoblotted with anti-ubiquitin antibody.

### Quantitative RT–PCR

Total RNA was extracted from cultured tumour cells dissolved in Isogen (Nippon Gene, Tokyo, Japan) and reverse transcribed at 37 °C for 1 h with SuperScript II reverse transcriptase (Invitrogen). The resultant cDNA was used for PCR amplification using Universal Probe Library System (Roche Diagnostics, Mannheim, Germany) using specific primers and a TaqMan probe on the LightCycler instrument (Roche Diagnostics) as described previously (Ito *et al*, 2005). GAPDH was analysed as an internal control. Sequences of primer pairs used in this study are listed in Supplementary Table 1S.

### Confocal laser scanning microscopic analysis

The cultured cells grown on coverslips were fixed in 4% paraformaldehyde in PBS and reacted with anti-EGFR antibody for 1 h. After rinsing, the primary antibody was detected with AlexaFluor 488-conjugated goat-anti-mouse IgG and nucleus was counterstained with Hoechst33324. The stained cells on cover slips were mounted on slide glass using Vectashield mounting medium (Vector Laboratories, Burlingame, CA, USA) and then viewed with a × 40 objective lens using a LSM510META confocal laser scanning microscope (Carl Zeiss, Jena, Germany).

### Animals

Seven-week-old male athymic nude mice of the KSN strain were purchased from the Shizuoka Laboratory Animal Center (Hamamatsu, Japan) and maintained under specific pathogen-free conditions. Animal experiments were carried out with the approval of the Institutional Ethical Committee for Animal Experiments of Aichi Cancer Center Research Institute and met the standard defined by the British Journal of Cancer guidelines (Workman *et al*, 2010).

### Immunohistochemical analysis

Subcutaneous tumours formed 1 month after injection of UMSCC81B-GR3 tumour cells into nude mice of KSN strain were removed and fixed in 10% buffered formalin for 24 h. Formalin-fixed and paraffin embedded sections (4 *μ*m) were used for immunohistochemistry as described previously (Yokoyama *et al*, 2006). Briefly, immunohistochemistry was performed using indirect immunoperoxidase method with first antibody to E-cadherin and vimentin, followed by incubation with biotinylated secondary antibody and subsequent incubation with streptavidin-peroxidase complex (Vectastain ABC kit, Vector Laboratories).

### Statistical analysis

The statistical significance of the differences in data between each treatment groups was determined by applying Student's *t*-test. A *P*-value 0.05 was considered significant.

## Results

### Isolation of gefitinib-resistant cells

**Figure 1:**
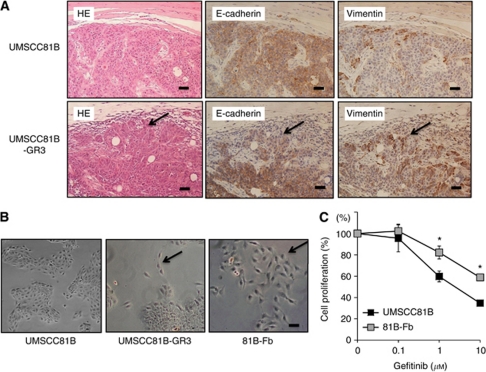
Isolation of fibroblastoid subline with EMT phenotype (81B-Fb) from gefitinib-resistant UMSCC81B-GR3 cell line obtained after long-term gefitinib treatment *in vitro*. (**A**) Immunohistochemistry of UMSCC81B-GR3 subcutaneous tumour in nude mouse. E-cadherin(−)/vimentin(+) tumour cells are seen at the invasion front of UMSCC81B-GR3 tumour (arrows). Bars=100 *μ*m. (**B**) Phase-contrast photomicrographs of cultured UMSCC81B, UMSCC81B-GR3 and fibroblastoid 81B-Fb cells. Arrow=fibroblastoid tumour cells. Bars=30 *μ*m. (**C**) Comparison of gefitinib sensitivity between UMSCC81B cells (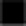) and 81B-Fb cells (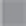). Bars= s.d., ^*^P<0.05.

We generated a gefitinib-resistant cell line (UMSCC81B-GR3) by repetitive gefitinib treatment from the parental HNSCC line (UMSCC81B). Immunohistochemical analysis of subcutaneous tumour formed after injection of UMSCC81B-GR3 cells into nude mice showed that E-cadherin(−)/vimentin(+) tumour cells that are less cohesive and exhibit a poorly-differentiated morphology proliferated at the invasion front of the tumour, but were almost absent in parental UMSCC81B tumour (Figure 1A). *In vitro* analysis showed that a small number of fibroblastoid variant tumour cells appeared around the epithelial cell nest of UMSCC81B-GR3 cells (Figure 1B arrows). By mechanical scraping of epithelial cells, pure fibroblastic tumour cell line (designated 81B-Fb) was successfully isolated (Figure 1B). This 81B-Fb cell line showed significantly lower sensitivity to gefitinib than parental cells with IC50 2.85 *vs* 30 *μ*M, respectively (Figure 1C). Such tumour cells undergoing EMT in the subcutaneous tumour are most likely the origin of 81B-Fb cells *in vitro*.

### Expression of EMT phenotype in 81B-Fb cells

**Figure 2:**
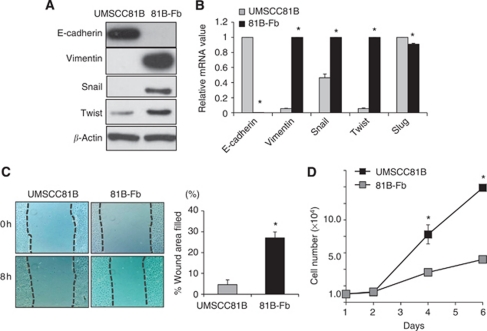
EMT phenotypic expression of 81B-Fb cells compared with parental UMSCC81B cells. (**A**) Western blot analysis of EMT-associated proteins. (**B**) mRNA expression of EMT-associated genes of UMSCC81B cells (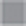) and 81B-Fb cells (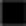). Loss of E-cadherin and acquisition of vimentin and snail expression are apparent. (**C**) Motility of UMSCC81B cells (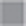) and 81B-Fb cells (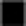) as measured by wound-closure assay. (**D**) *In vitro* growth rate of UMSCC81B cells (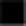) and 81B-Fb cells (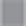). Bars=s.d., ^*^P<0.05.

There was a remarkable change from an epithelial to a fibroblast-like morphology in 81B-Fb cells. This finding prompted us to examine EMT-associated phenotypes of 81B-Fb cells. We first examined expression of EMT-associated molecules. In western blotting, loss of E-cadherin, acquisition of vimentin and snail expression and increase in twist expression were clearly observed in 81B-Fb cells compared with UMSCC81B cells (Figure 2A). Quantitative RT–PCR analysis confirmed almost complete loss of E-cadherin and upregulation of vimentin, snail and twist, but not slug, in 81B-Fb cells compared with UMSCC81B cells (Figure 2B). We then conducted wound-closure assays for measuring motility of these cells. As shown in Figure 2C, migration ability of 81B-Fb cells is significantly increased compared with UMSCC81B cells. However, *in vitro* growth rate of 81B-Fb cells is significantly slower than UMSCC81B cells (Figure 2D). Similar, but partial acquisition of EMT phenotype was observed in another HNSCC cell line (HSC3) after repetitive gefitinib treatment *in vitro* (Supplementary Figure 1S).

### Downregulation and cytoplasmic localisation of EGFR in 81B-Fb cells

**Figure 3:**
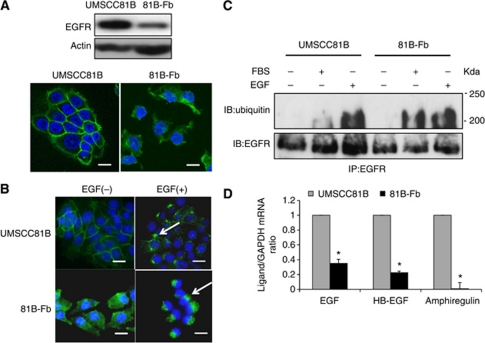
Downregulation of EGFR expression in 81B-Fb cells compared with UMSCC81B cells. (**A**) Western blot and immunofluorescence analysis of EGFR protein expression of 81B-Fb cells. Change of subcellular localisation of EGFR from membrane to the cytoplasm is apparent in 81B-Fb cells in the presence of FBS. (**B**) Internalisation of EGFR in UMSCC81B and 81B-Fb cells in response to EGF. Cells were serum-starved for 24 h, and stimulated with EGF (20 ng ml^−1^). Accumulation of EGFR in the endosome (arrows) is seen in both cells at 15 min after EGF stimulation. (**C**) Immunoprecipitation analysis of ubiquitination of EGFR in response to FBS or EGF. Cells were serum-starved for 24 h, pretreated with 10 mM MG132 for 2 h and then stimulated with EGF for 15 min or FBS for several hours. (**D**) mRNA expression of various EGFR ligands of UMSCC81B cells (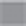) and 81B-Fb cells (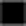). Bars=s.d., ^*^P<0.05.

Western blotting showed that EGFR protein expression was downregulated in 81B-Fb cells compared with UMSCC81B cells. Consistent with this, immunofluorescence microscopy revealed that subcellular localisation of EGFR changed from plasma membrane in UMSCC81B cells to almost cytoplasm in 81B-Fb cells in the presence of FBS (Figure 3A). Stimulation of serum-starved UMSCC81B cells with EGF ligand resulted in the internalisation of EGFR from plasma membrane-like 81B-Fb cells. However, upon stimulation with ligand, EGFR accumulated in the endosome, a more specific area, in both UMSCC81B and 81B-Fb cells (Figure 3B). As the internalisation of EGFR after EGF stimulation is known to be mediated by ubiquitination, we next compared ubiquitination of EGFR in UMSCC81B cells and 81B-Fb cells by immunoprecipitaion. Upon stimulation with EGF, EGFR was polyubiquitinated in both cells to the same extent. In contrast, ubiquitination of EGFR was significantly higher in 81B-Fb than in UMSCC81B cells in the presence of FBS (Figure 3C), consistent with downregulation and internalisation of EGFR in 81B-Fb cells. To examine the possibility of increased EGFR internalisation in 81B-Fb cells via autocrine stimulation with EGF, we measured mRNA for various ligands for EGFR such as EGF, HB-EGF and amphiregulin. Expression of all these ligands was significantly lower in 81B-Fb cells than in parental cells, suggesting that downregulation and internalisation of EGFR seen in 81B-Fb cells is not caused by enhanced ubiquitination through autocrine stimulation by the EGF ligand (Figure 3D).

### Effects of gefitinib on phosphorylation of EGFR and downstream signalling

**Figure 4:**
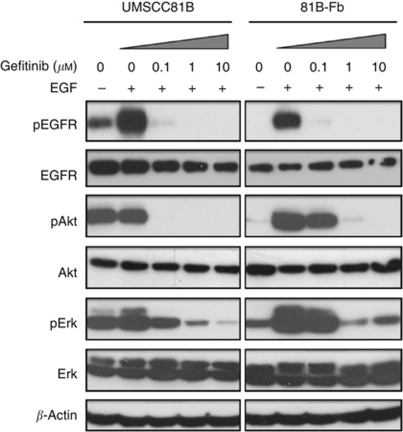
Western blot analysis of the effects of gefitinib on the phosphorylation of EGFR and downstream signalling pathways in 81B-Fb cells compared with UMSCC81B cells. Cells were starved in serum-free medium for 24 h, exposed to gefitinib at increasing concentrations for 2 h and were then stimulated with EGF (10 ng ml^−1^) for 10 min.

To investigate the mechanism of acquired gefitinib resistance in 81B-Fb cells, we compared activation of EGFR and downstream signalling between the two cells. In serum-starved 81B-Fb cells, total EGFR and phosphorylated EGFR, Akt and Erk were lower than UMSCC81B cells in the absence of EGF. However, the EGF-induced increase in phosphorylation of Akt and Erk was higher and more resistant to inhibition by gefitinib, especially for Akt, in the 81B-Fb cells than in parental cells (Figure 4), indicating more gefitinib resistance of 81B-Fb cells.

### Involvement of Akt/GSK-3*β*/snail pathway in the acquisition of EMT in 81B-Fb cells

**Figure 5:**
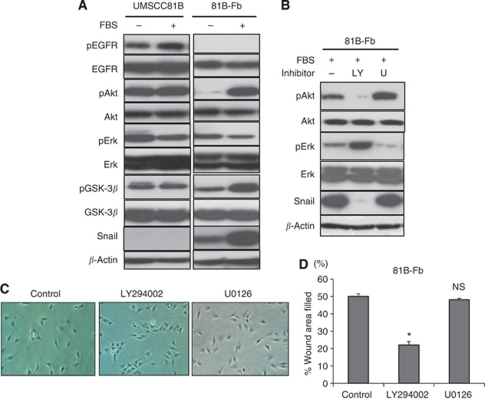
Effects of PI3K inhibitor and MEK1/2 inhibitor on the signalling pathway and EMT phenotypes in 81B-Fb cells. (**A**) Activation of Akt/GSK-3β and upregulation of snail expression in response to FBS in 81B-Fb cells compared with UMSCC81B cells. (**B**) Inactivation of Akt and downregulation of snail by LY294002 in 81B-Fb cells. Cells were cultured in DMEM with 10% FBS and exposed each inhibitors for 12 h. LY: LY294002 (50 *μ*M), U: U0126 (20 *μ*M). (**C** and **D**) Effects of signal inhibitors on the morphology (**C**) and motility (**D**) of 81B-Fb cells. LY294002 (25 *μ*M) but not U0126 (10 *μ*M) disturbs fibroblastoid morphology and inhibits migration of 81B-Fb cells. Bars=s.d., ^*^P<0.05. Abbreviation: NS=not significant.

In UMSCC81B cells, EGFR and downstream signalling such as Akt/GSK-3*β* and Erk were constitutively activated in the absence of FBS and EGF, whereas in 81B-Fb cells, only Erk was activated. Upon stimulation with FBS, phosphorylation of Akt and GSK-3*β* was enhanced and snail expression was upregulated in 81B-Fb cells, suggesting the possibility that Akt/GSK-3*β* pathway is involved in the regulation of snail expression (Figure 5A). Therefore, we next examined whether snail expression of 81B-Fb cells is inhibited by either LY294002 or U0126. As a result, only LY294002 significantly inhibited snail expression of 81B-Fb cells in line with the inactivation of Akt (Figure 5B). Fibroblastic morphology and motility were also significantly inhibited by LY294002 but not U0126 (Figure 5C and D).

### Partial restoration of epithelial phenotype and gefitinib sensitivity by the EGFR transfection in 81B-Fb cells

**Figure 6:**
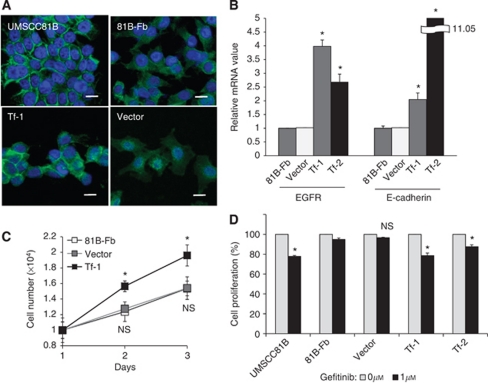
Effects of EGFR transfection on the growth, differentiation and gefitinib sensitivity of 81B-Fb cells. (**A**) Subcellular localisation of EGFR in the UMSCC81B cells, 81B-Fb cells, EGFR transfectant of 81B-Fb cells (Tf-1) and vector control. Note, restoring membrane expression of EGFR in Tf-1 cells compared with 81B-Fb cells and vector control cells. (**B**) Quantitative RT–PCR analysis. E-cadherin mRNA expression is significantly increased in EGFR transfectants in accordance with increased EGFR expression. (**C**) *In vitro* growth of 81B-Fb cells (□), EGFR transfectant (Tf-1; 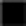) and vector control (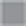). (**D**) Comparison of gefitinib sensitivity among various tumour cells with (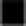) and without gefitinib (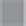). Bars=s.d., ^*^P<0.05. Abbreviation: NS=not significant.

To clarify the role of downregulation and internalisation of EGFR in the induction of EMT, we conducted a transfection experiment with EGFR plasmid. In the stable transfectants (Tf-1 and Tf-2), EGFR and E-cadherin expression increased at the extent of approximately 2- to 4-fold and 3- to 11-fold at the mRNA level, respectively (Figure 6B). In accordance with such increased EGFR expression, membrane localisation of EGFR was partially restored in these transfectants compared with 81B-Fb cells and vector control cells (Figure 6A). Forced expression of EGFR also resulted in the partial recovery of growth potential and gefitinib sensitivity (Figure 6C and D), indicating that at least in part, EGFR downregulation and internalisation are responsible for both generation of EMT and acquisition of gefitinib resistance.

### Expression of HER receptor family and neuregulin ligands in UMSCC81B and 81B-Fb cells

To explore the potential mechanism of Akt/GSK-3*β* pathway activation in 81B-Fb cells, we compared expression of various HER family receptors and neuregulin ligands between UMSCC81B and 81B-Fb cells. Western blotting analysis showed that EGFR is moderately and HER3 is markedly downregulated, whereas HER2 is compensatory upregulated in the 81B-Fb cells compared with UMSCC81B cells. In addition, among ligands for HER3, neuregulin 1 (NRG1) is markedly reduced, but NRG2 is significantly upregulated in 81B-Fb cells compared with UMSCC81B cells (Supplementary Figure 3S).

In relation to this upregulation of HER2, we further examined the effects of HER2 inhibitors such as lapatinib and trastuzumab on the EMT phenotypes to investigate the possible role of HER2 in the induction of gefitinib resistance and EMT. The results showed that lapatinib only weakly inhibited growth, EMT and Akt/GSK-3*β*/snail signalling compared with gefitinib or LY294002, and trastuzumab had no effect on the growth and EMT (Supplementary Figure 4S).

## Discussion

In the present study, we newly isolated a gefitinib-resistant variant subline from UMSCC81B parent cell line by repetitive, dose-escalating gefitinib treatment *in vitro*. Interestingly, this gefitinib-resistant variant line (81B-Fb) consists of only fibroblast-like tumour cells and shows typical characteristics of EMT such as almost complete loss of E-cadherin, increased vimentin and snail expression and increased cell motility. Immunohistochemical analysis of transplanted tumour suggests that such 81B-Fb cells are originated from E-cadherin(−)/vimentin(+) tumour cells present at the invasion front of UMSCC81B-GR3 tumour tissue. Emergence of gefitinib-resistant cell line with a similar but modest EMT-like phenotype such as vimentin expression without obvious fibroblastic morphology after repetitive gefitinib treatment was also observed in another HNSCC line, HSC3 (Supplementary Figure 1S), but not in HSC2 and UMSCC6 cell lines. Epithelial mesenchymal transition-inducible UMSCC81B and HSC3 cell lines are histologically poorly-differentiated HNSCC lines, whereas EMT non-inducible HSC2 and UMSCC6 lines are well-differentiated keratinising HNSCC cell lines, suggesting that the HNSCC cell line harbouring a partial EMT-like phenotype, such as simultaneous E-cadherin and vimentin expression, has a potential for generating fully dedifferentiated EMT. Several investigators reported the emergence of EMT by treatment with chemotherapeutic agents such as gemcitabine and adriamycin in pancreatic and breast cancer cell lines, respectively (Li *et al*, 2009; Wang *et al*, 2009). Morgillo *et al* also reported that NSCLC cell line with resistance to TKI exhibited EMT-like phenotype (Morgillo *et al*, 2011). To our knowledge, however, this is the first EMT line of HNSCC with resistance to EGFR-targeting agent and would thus offer a useful *in vitro* model to understand the mechanism underlying the link between EMT and gefitinib resistance.

Using this EMT model, we investigated the mechanism by which EMT emerges in the HNSCC cell lines after repetitive gefitinib treatment. We found that simultaneous upregulation of Akt/GSK-3*β* and snail occurred in response to FBS in 81B-Fb cells and that such activation of Akt and snail overexpression as well as cell motility of 81B-Fb cells in the presence of FBS was effectively inhibited by LY294002 but not U0126. Akt reportedly induces inactivation (phosphorylation) of GSK-3*β*, which in turn suppresses phosphorylation of snail to induce the nuclear localisation and protein stabilisation of snail, leading to EMT (Zhou *et al*, 2004; Touny and Banerjee, 2007). Participation of Akt/GSK-3*β***/**snail pathway in the EMT has also been reported previously in hepatocellular carcinoma line (Assinder *et al*, 2009; Wu *et al*, 2011). These findings strongly suggest that Akt/GSK-3*β***/**snail pathway is involved in the induction of EMT phenotype in 81B-Fb cells. As PI3K mutation and PTEN loss were not observed in 81B-Fb cells (data not shown), aberrant ligand-receptor binding may be responsible for the activation of downstream Akt/GSK-3*β***/**snail signalling. There are at least three explanations for this. The first candidate is EGF, which is known to be able to induce EMT phenotype in several cancer cell lines including HNSCC (Zuo *et al*, 2011). In fact, EGF activated the Akt/GSK-3*β* pathway in 81B-Fb cells, but the degree of snail upregulation by EGF was weaker than that by FBS (data not shown). Moreover, EGF itself is rather downregulated in 81B-Fb cells, suggesting that unknown factors (ligand) present in the FBS other than EGF are mainly responsible for the activation of Akt/GSK-3*β***/**snail pathway in 81B-Fb cells. The second candidate ligand present in FBS is TGF-*β* which is also known to mediate EMT via canonical TGF-*β*/smad/snail signalling pathway or non-canonical TGF-*β*/Akt/GSK-3*β***/**snail signalling pathway (Vincent *et al*, 2009). We found that TGF-*β* receptors (T*β*RI and T*β*RII) were upregulated and downstream Smad3 was more phosphorylated in the 81B-Fb cells than UMSCC81B cells (data not shown). However, Akt/GSK-3*β*/snail pathway and cell motility were not significantly affected by treatment with TGF-*β* or TGF*β*RI inhibitor, SD208 (Supplementary Figure 2S). Therefore, the possibility of direct regulation of snail expression by non-canonical TGF-*β*/Akt/GSK-3*β***/**snail signalling pathway for 81B-Fb cells is unlikely, although involvement of canonical TGF-*β*/Smad/snail pathway in snail expression still remains to be determined. The third possibility is the activation of an HER2 ligand/receptor family (Quesnelle and Grandis, 2011). We found that EGFR and HER3 are downregulated, whereas HER2 is compensatorily upregulated in the 81B-Fb cells compared with UMSCC81B cells. In addition, neuregulin 2 (NRG2), but not NRG1, which are ligands for HER3, is upregulated in 81B-Fb cells compared with UMSCC81B cells (Supplementary Figure 3S). These results suggest the possibility that the activation of Akt/GSK-3*β***/**snail signalling pathway observed in 81B-Fb cells is mediated by the NRG2-induced HER2/HER3 heterodimer formation (Carraway *et al*, 1997; Hobbs *et al*, 2002). We speculate that such HER2 overexpression, in compensation with downregulated EGFR, has a role in the activation of Akt/GSK-3*β*/snail pathway, although HER2 inhibitors such as lapatinib did not exhibit significant inhibitory effect on EMT unlike PI3K inhibitor, probably due to its multi functional effects on the various signalling pathways (Supplementary Figure 4S). Regardless of such upstream signalling at receptor level, the fact that downstream Akt/GSK-3*β*/snail pathway is involved in the induction of EMT in 81B-Fb cell suggests the possibility that molecular agents targeting the Akt/GSK-3*β* signalling pathway rather than HER2 is a promising approach to overcome EMT. Further study is needed to clarify the detailed molecular mechanism of activation of the Akt/GSK-3*β*/snail pathway in 81B-Fb cells.

Another important objective in this study was to clarify the mechanism of gefitinib resistance in 81B-Fb cells. We found that EGFR is downregulated and subcellular localisation of EGFR changed from plasma membrane to cytoplasm in 81B-Fb cells. This downregulation of EGFR is probably mediated by enhanced ubiquitination of EGFR and subsequent proteosomal degradation as reported previously (Lu *et al*, 2007). Ligand (EGF)-induced phosphorylation of downstream Akt and Erk in the 81B-Fb cells were much higher and more resistant to inhibition by gefitinib than UMSCC81B cells. Furthermore, forced expression of EGFR on the cell surface membrane partially reversed sensitivity of 81B-Fb cells to gefitinib. Incidentally, EGFR mutations such as T790M and MET amplification reported in NSCLC (Kosaka *et al*, 2006; Cappuzzo *et al*, 2009) as a cause of gefitinib resistance were not observed in 81B-Fb cells (data not shown). These results suggest that gefitinib resistance of 81B-Fb cells is mediated principally by downregulation of EGFR. Further study is needed to clarify the detailed mechanism of ubiquitin-mediated EGFR downregulation.

In conclusion, we isolated a new EMT model from HNSCC line with gefitinib resistance and demonstrated that EMT as well as gefitinib resistance is mediated by the downregulation of membrane EGFR through compensatory activation of Akt/GSK-3*β*/snail pathway. We have experienced recurrent cases of a more aggressive tumour with EMT phenotype after cetuximab in combination with radiation therapy in patients with HNSCC. Therefore, it is clinically very important to prevent drug-induced EMT with resistance to EGFR-targeting therapy. The present EMT line would thus offer an excellent model that will lead to the detailed analysis of the underlying mechanism and thereby development of new effective therapy for EGFR targeting drug-resistant HNSCC with EMT phenotype.

## References

[bib1] Assinder SJ, Dong Q, Kovacevic Z, Richardson DR (2009) The TGF-beta, PI3K/Akt and PTEN pathways: established and proposed biochemical integration in prostate cancer. Biochem J 417: 411–421 19099539 10.1042/BJ20081610

[bib2] Cappuzzo F, Janne PA, Skokan M, Finocchiaro G, Rossi E, Ligorio C, Zucali PA, Terracciano L, Toschi L, Roncalli M, Destro A, Incarbone M, Alloisio M, Santoro A, Varella-Garcia M (2009) MET increased gene copy number and primary resistance to gefitinib therapy in non-small-cell lung cancer patients. Ann Oncol 20: 298–304 18836087 10.1093/annonc/mdn635 PMC2733067

[bib3] Carraway III KL, Weber JL, Unger MJ, Ledesma J, Yu N, Gassmann M, Lai C (1997) Neuregulin-2 a new ligand of ErbB3/ErbB4-receptor tyrosine kinases. Nature 387: 512–516 9168115 10.1038/387512a0

[bib4] Chen HJ, Mok TS, Chen ZH, Guo AL, Zhang XC, Su J, Wu YL (2009) Clinicopathologic and molecular features of epidermal growth factor receptor T790M mutation and c-MET amplification in tyrosine kinase inhibitor-resistant Chinese non-small cell lung cancer. Pathol Oncol Res 15: 651–658 19381876 10.1007/s12253-009-9167-8

[bib5] Erjala K, Sundvall M, Junttila TT, Zhang N, , Savisalo M, , Mali P, Kulmala J, Pulkkinen J, Grenman R, Elenius K (2006) Signaling via ErbB2 and ErbB3 associates with resistance and epidermal growth factor receptor (EGFR) amplification with sensitivity to EGFR inhibitor gefitinib in head and neck squamous cell carcinoma cells. Clin Cancer Res 12: 4103–4111 16818711 10.1158/1078-0432.CCR-05-2404

[bib6] Frederick BA, Helfrich BA, Coldren CD, Zheng D, Chan D, Bunn Jr PA, Raben D (2007) Epithelial to mesenchymal transition predicts gefitinib resistance in cell lines of head and neck squamous cell carcinoma and non-small cell lung carcinoma. Mol Cancer Ther 6: 1683–1691 17541031 10.1158/1535-7163.MCT-07-0138

[bib7] Hama T, Yuza Y, Saito Y, O-uchi J, Kondo S, Okabe M, Yamada H, Kato T, Moriyama H, Kurihara S, Urashima M (2009) Prognostic significance of epidermal growth factor receptor phosphorylation and mutation in head and neck squamous cell carcinoma. Oncologist 14: 900–908 19726454 10.1634/theoncologist.2009-0058

[bib8] Hobbs SS, Coffing SL, Le AT, Cameron EM, Williams EE, Andrew M, Blommel EN, Hammer RP, Chang H, Riese II DJ (2002) Neuregulin isoforms exhibit distinct patterns of ErbB family receptor activation. Oncogene 21: 8442–8452 12466964 10.1038/sj.onc.1205960

[bib9] Ito S, Nakanishi H, Kodera Y, Mochizuki Y, Tatematsu M, Yamamura Y (2005) Prospective validation of quantitative CEA mRNA detection in peritoneal washes in gastric carcinoma patients. Br J Cancer 93: 986–992 16205696 10.1038/sj.bjc.6602802 PMC2361668

[bib10] Iwatsuki M, Mimori K, Yokobori T, Ishi H, Beppu T, Nakamori S, Baba H, Mori M (2010) Epithelial-mesenchymal transition in cancer development and its clinical significance. Cancer Sci 101: 293–299 19961486 10.1111/j.1349-7006.2009.01419.x PMC11159985

[bib11] Kalyankrishna S, Grandis JR (2006) Epidermal growth factor receptor biology in head and neck cancer. J Clin Oncol 24: 2666–2672 16763281 10.1200/JCO.2005.04.8306

[bib12] Klymkowsky MW, Savagner P (2009) Epithelial-mesenchymal transition: a cancer researcher's conceptual friend and foe. Am J Pathol 174: 1588–1593 19342369 10.2353/ajpath.2009.080545 PMC2671246

[bib13] Kosaka T, Yatabe Y, Endoh H, Yoshida K, Hida T, Tsuboi M, Tada H, Kuwano H, Mitsudomi T (2006) Analysis of epidermal growth factor receptor gene mutation in patients with non-small cell lung cancer and acquired resistance to gefitinib. Clin Cancer Res 12: 5764–5769 17020982 10.1158/1078-0432.CCR-06-0714

[bib14] Li QQ, Xu JD, Wang WJ, Cao XX, Chen Q, Tang F, Chen ZQ, Liu XP, Xu ZD (2009) Twist1-mediated adriamycin-induced epithelial-mesenchymal transition relates to multidrug resistance and invasive potential in breast cancer cells. Clin Cancer Res 15: 2657–2665 19336515 10.1158/1078-0432.CCR-08-2372

[bib15] Lu Y, Li X, Liang K, Luwor R, Siddik ZH, Mills GB, Mendelsohn J, Fan Z (2007) Epidermal growth factor receptor (EGFR) ubiquitination as a mechanism of acquired resistance escaping treatment by the anti-EGFR monoclonal antibody cetuximab. Cancer Res 67: 8240–8247 17804738 10.1158/0008-5472.CAN-07-0589

[bib16] Morgillo F, Cascone T, D'Aiuto E, Martinelli E, Troiani T, Saintigny P, De Palma R, Heymach JV, Berrino L, Tuccillo C, Ciardiello F (2011) Antitumour efficacy of MEK inhibitors in human lung cancer cells and their derivatives with acquired resistance to different tyrosine kinase inhibitors. Br J Cancer 105: 382–392 21750552 10.1038/bjc.2011.244 PMC3172903

[bib17] Nozawa H, Tadakuma T, Ono T, Sato M, Hiroi S, Masumoto K, Sato Y (2006) Small interfering RNA targeting epidermal growth factor receptor enhances chemosensitivity to cisplatin, 5-fluorouracil and docetaxel in head and neck squamous cell carcinoma. Cancer Sci 97: 1115–1124 16984384 10.1111/j.1349-7006.2006.00287.x PMC11158321

[bib18] Quesnelle KM, Grandis JR (2011) Dual kinase inhibition of EGFR and HER2 overcomes resistance to cetuximab in a novel *in vivo* model of acquired cetuximab resistance. Clin Cancer Res 17: 5935–5944 21791633 10.1158/1078-0432.CCR-11-0370 PMC3426303

[bib19] Sok JC, Coppelli FM, Thomas SM, Lango MN, Xi S, Hunt JL, Freilino ML, Graner MW, Wikstrand CJ, Bigner DD, Gooding WE, Furnari FB, Grandis JR (2006) Mutant epidermal growth factor receptor (EGFRvIII) contributes to head and neck cancer growth and resistance to EGFR targeting. Clin Cancer Res 12: 5064–5073 16951222 10.1158/1078-0432.CCR-06-0913

[bib20] Stewart JS, Cohen EE, Licitra L, Van Herpen CM, Khorprasert C, Soulieres D, Vodvarka P, Rischin D, Garin AM, Hirsch FR, Varella-Garcia M, Ghiorghiu S, Hargreaves L, Armour A, Speake G, Swaisland A, Vokes EE (2009) Phase III study of gefitinib compared with intravenous methotrexate for recurrent squamous cell carcinoma of the head and neck. J Clin Oncol 27: 1864–1871 19289630 10.1200/JCO.2008.17.0530

[bib21] Touny LH, Banerjee PP (2007) Akt GSK-3 pathway as a target in genistein-induced inhibition of TRAMP prostate cancer progression toward a poorly differentiated phenotype. Carcinogenesis 28: 1710–1717 17468512 10.1093/carcin/bgm103

[bib22] Uramoto H, Iwata T, Onitsuka T, Shimokawa H, Hanagiri T, Oyama T (2010) Epithelial-mesenchymal transition in EGFR-TKI acquired resistant lung adenocarcinoma. Anticancer Res 30: 2513–2517 20682976

[bib23] Vermorken JB, Mesia R, Rivera F, Remenar E, Kawecki A, Rottey S, Erfan J, Zabolotnyy D, Kienzer HR, Cupissol D, Peyrade F, Benasso M, Vynnychenko I, De Raucourt D, Bokemeyer C, Schueler A, Amellal N, Hitt R (2008) Platinum-based chemotherapy plus cetuximab in head and neck cancer. N Engl J Med 359: 1116–1127 18784101 10.1056/NEJMoa0802656

[bib24] Vincent T, Neve EP, Johnson JR, Kukalev A, Rojo F, Albanell J, Pietras K, Virtanen I, Philipson L, Leopold PL, Crystal RG, de Herreros AG, Moustakas A, Pettersson RF, Fuxe J (2009) A SNAIL1-SMAD3/4 transcriptional repressor complex promotes TGF-beta mediated epithelial-mesenchymal transition. Nat Cell Biol 11: 943–950 19597490 10.1038/ncb1905 PMC3769970

[bib25] Wang Z, Li Y, Kong D, Banerjee S, Ahmad A, Azmi AS, Ali S, Abbruzzese JL, Gallick GE, Sarkar FH (2009) Acquisition of epithelial-mesenchymal transition phenotype of gemcitabine-resistant pancreatic cancer cells is linked with activation of the notch signaling pathway. Cancer Res 69: 2400–2407 19276344 10.1158/0008-5472.CAN-08-4312 PMC2657919

[bib26] Workman P, Aboagye EO, Balkwill F, Balmain A, Bruder G, Chaplin DJ, Double JA, Everitt J, Farningham DA, Glennie MJ, Kelland LR, Robinson V, Stratford IJ, Tozer GM, Watson S, Wedge SR, Eccles SA (2010) Guidelines for the welfare and use of animals in cancer research. Br J Cancer 102: 1555–1577 20502460 10.1038/sj.bjc.6605642 PMC2883160

[bib27] Wu J, Ru NY, Zhang Y, Li Y, Wei D, Ren Z, Huang XF, Chen ZN, Bian H (2011) HAb18G/CD147 promotes epithelial-mesenchymal transition through TGF-beta signaling and is transcriptionally regulated by Slug. Oncogene 30(43): 4410–4427 21532623 10.1038/onc.2011.149

[bib28] Yokoyama H, Ikehara Y, Kodera Y, Ikehara S, Yatabe Y, Mochizuki Y, Koike M, Fujiwara M, Nakao A, Tatematsu M, Nakanishi H (2006) Molecular basis for sensitivity and acquired resistance to gefitinib in HER2-overexpressing human gastric cancer cell lines derived from liver metastasis. Br J Cancer 95: 1504–1513 17088902 10.1038/sj.bjc.6603459 PMC2360749

[bib29] Zhou BP, Deng J, Xia W, Xu J, Li YM, Gunduz M, Hung MC (2004) Dual regulation of snail by GSK-3beta-mediated phosphorylation in control of epithelial-mesenchymal transition. Nat Cell Biol 6: 931–940 15448698 10.1038/ncb1173

[bib30] Zuo JH, Zhu W, Li MY, Li XH, Yi H, Zeng GQ, Wan XX, He QY, Li JH, Qu JQ, Chen Y, Xiao ZQ (2011) Activation of EGFR promotes squamous carcinoma SCC10A cell migration and invasion via inducing EMT-like phenotype change and MMP-9-mediated degradation of E-cadherin. J Cell Biochem 112: 2508–2517 21557297 10.1002/jcb.23175

