# Supplementary material for: Acquisition of EMT phenotype in the gefitinib-resistant cells of a head and neck squamous cell carcinoma cell line through Akt/GSK-3β/snail signalling pathway
Source: Br J Cancer. 2012 Feb 7;106(6):1196–204. doi: 10.1038/bjc.2012.24 (PMC3304404; doi:10.1038/bjc.2012.24)
Supplement: Supplementary Table and Figures [file bjc201224x1.pdf]

Supplementary Figure 1S

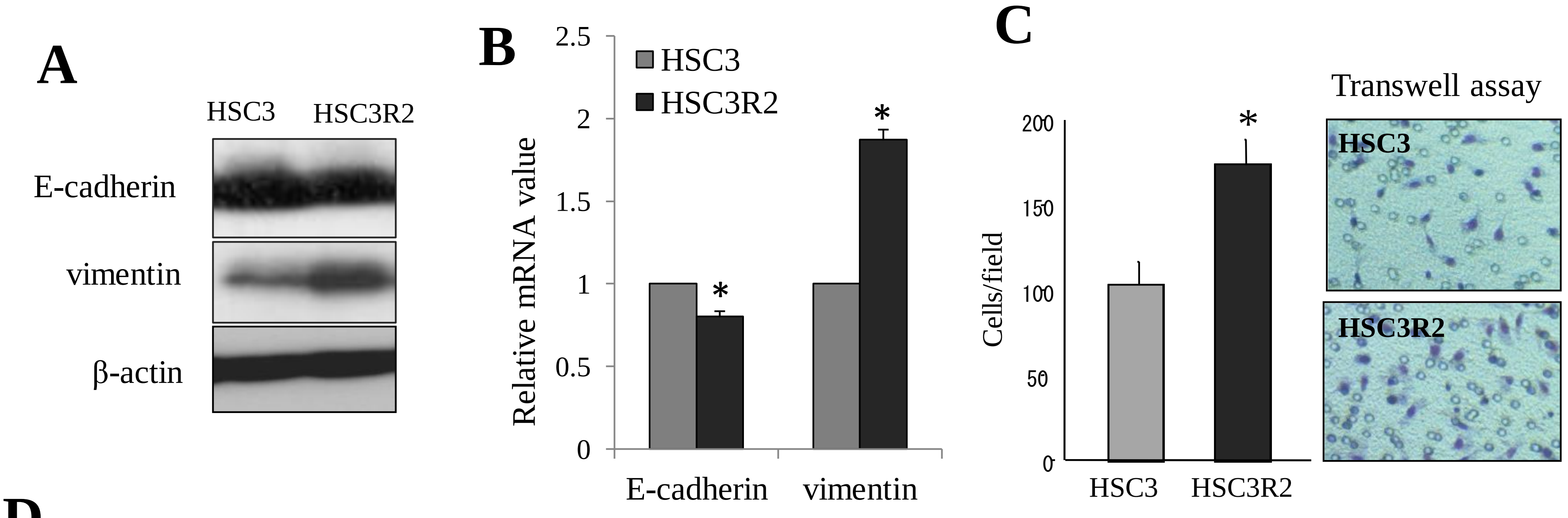

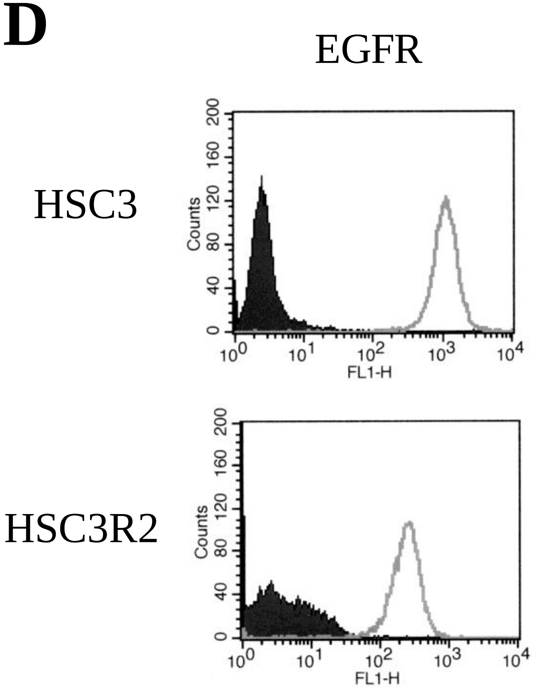

Figure 1S EMT phenotypic expression of HSC3R2 cells compared with parental HSC3 cells. **(A)** Western blot analysis of EMT-associated proteins. **(B)** mRNA expression of EMT-associated genes of HSC3 (■) cells and HSC3R2 cells (■). Increased vimentin expression in HSC3R2 cells is apparent. **(C)** Motility of HSC3 cells (■) and HSC3R2 cells (■) as measured by transwell migration assay. (D) Flow cytometric analysis of EGFR expression. Isotype control Ig (black) and anti EGFR antibody (gray), Bars= SD, *P<0.05

Supplementary Figure 2S

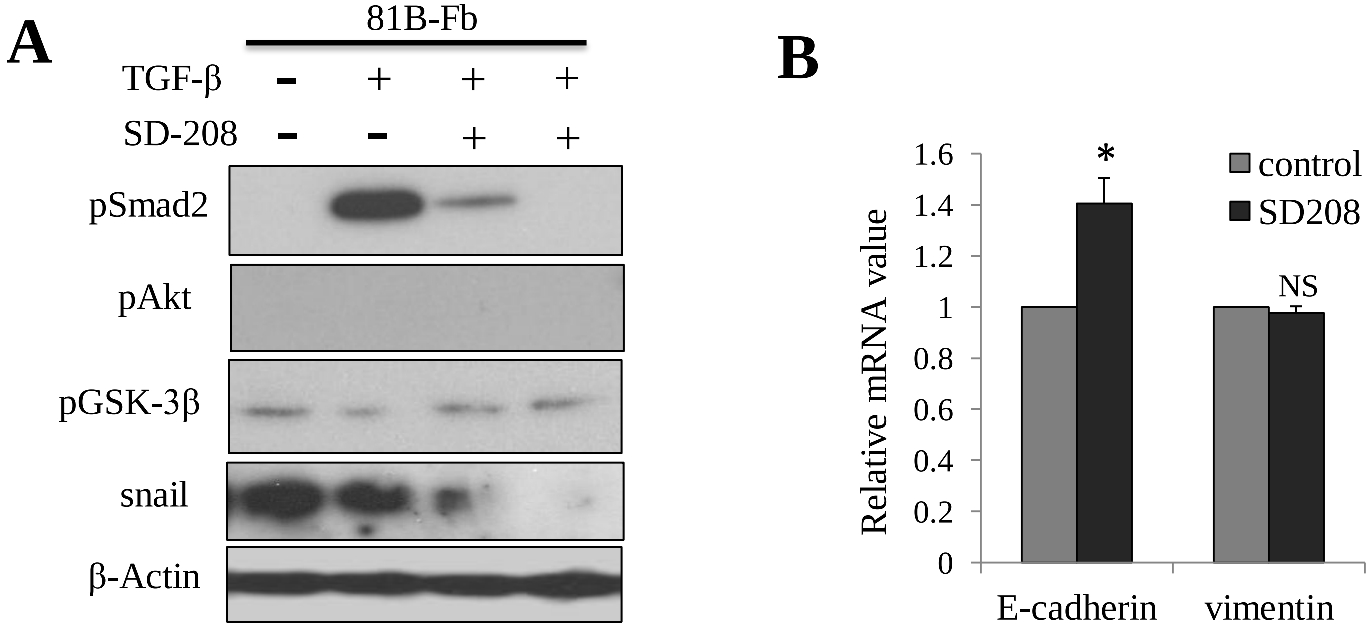

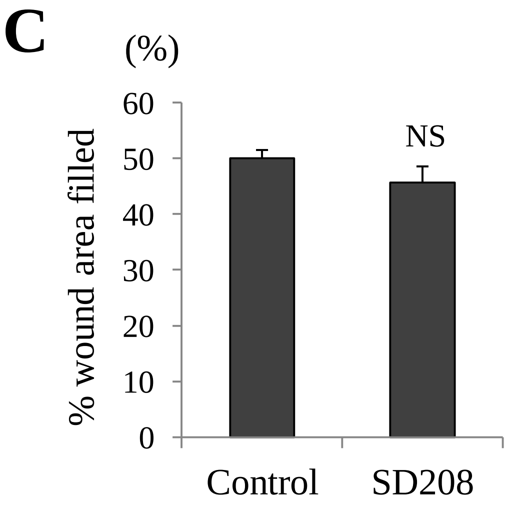

Figure 2S Effects of TGF-β and TGF-βRI inhibitor (SD208) on the EMT phenotype of 81B-Fb cells. (A) Western blot analysis. Cells were starved in serum-free medium for 24 h, and exposed to SD208 at increasing concentrations (1 and 5 μM) for 2 h at 37°C. Cells were then stimulated with TGF-β (2.5 ng/ml) for 10 min, lysed and subjected to Western blotting. (B) mRNA expression of E-cadherin and vimentin of 81B-Fb cells with or without SD208. (C) Motility of 81B-Fb cells as measured by wound-closure assay. Bars= SD, *$P<0.05$. NS=not significant.

Supplementary Figure 3S

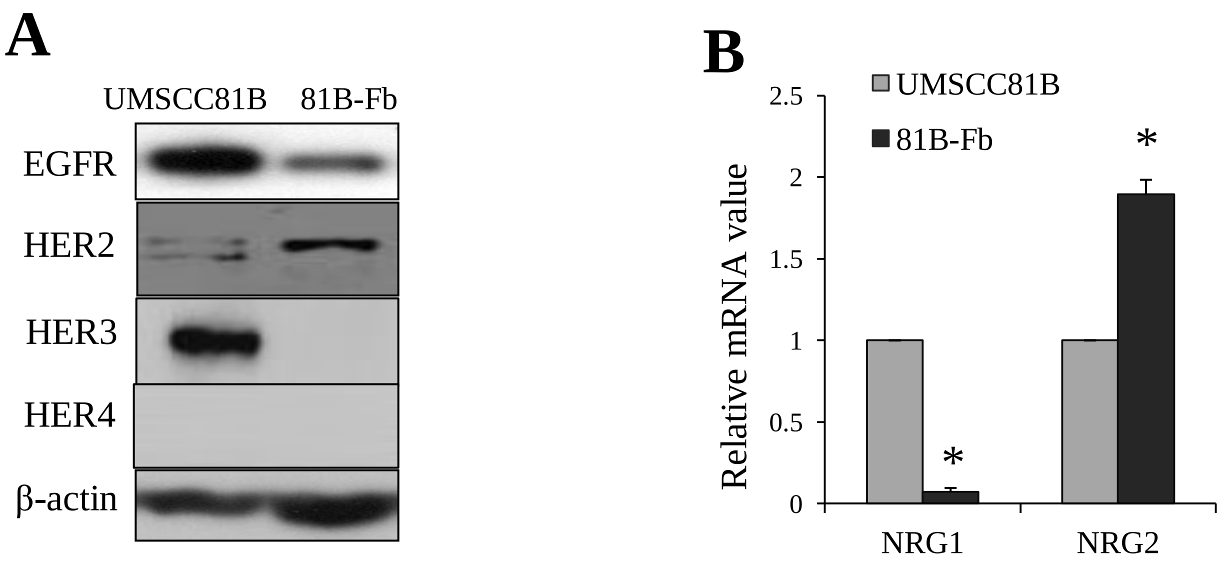

Figure 3S Expression of HER receptor family and neuregulin ligands in UMSCC81B and 81B-Fb cells. **(A)** Western blot analysis of EGFR, HER2, HER3 and HER4 in UMSCC81B and 81B-Fb cells. **(B)** qRT-PCR analysis of NRG1 and NRG2 mRNA expression in UMSCC81B cells (■) and 81B-Fb cells (■). Bars=SD, *P<0.05.

Supplementary Figure 4S

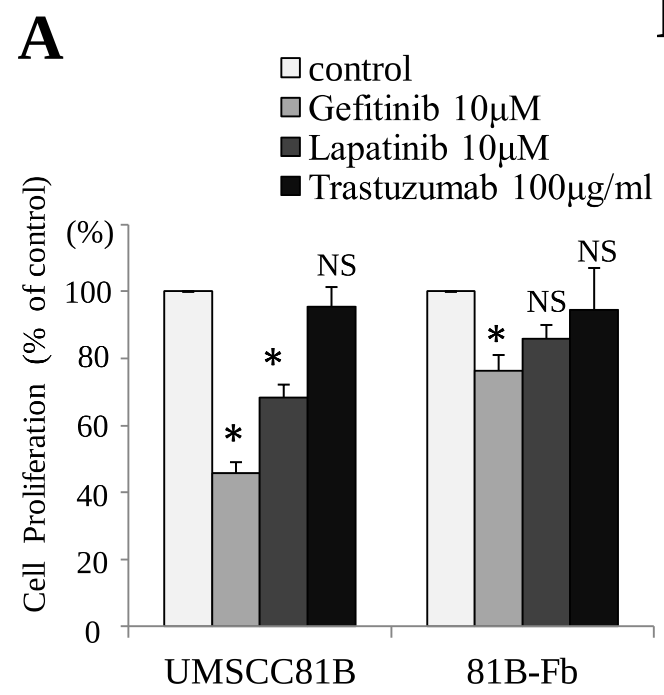

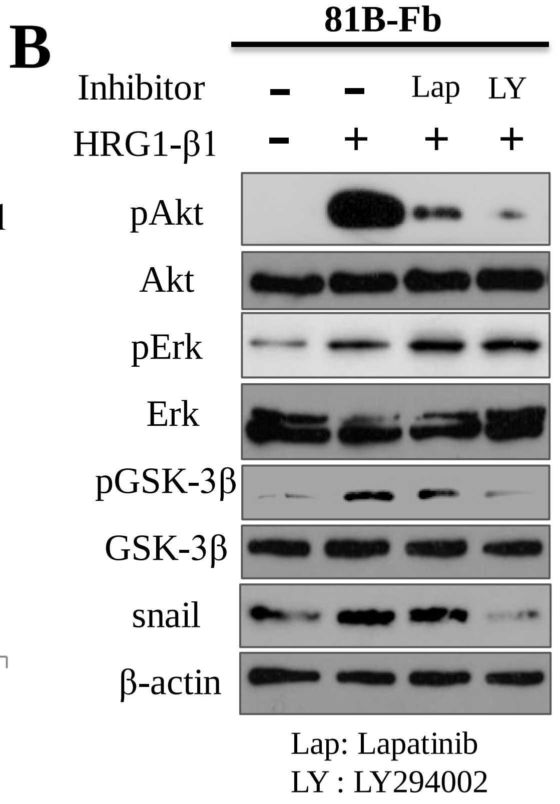

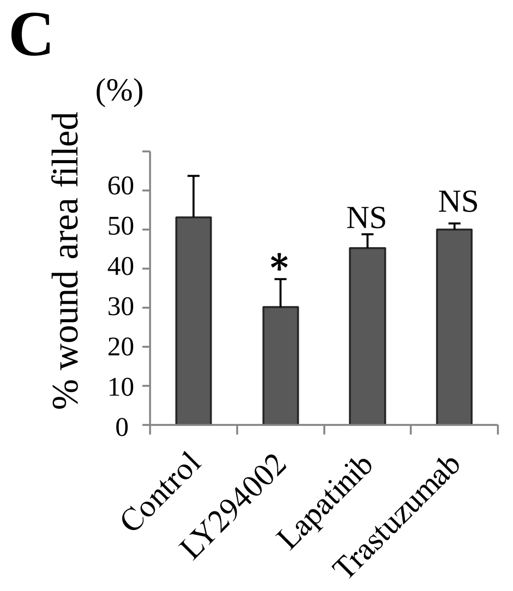

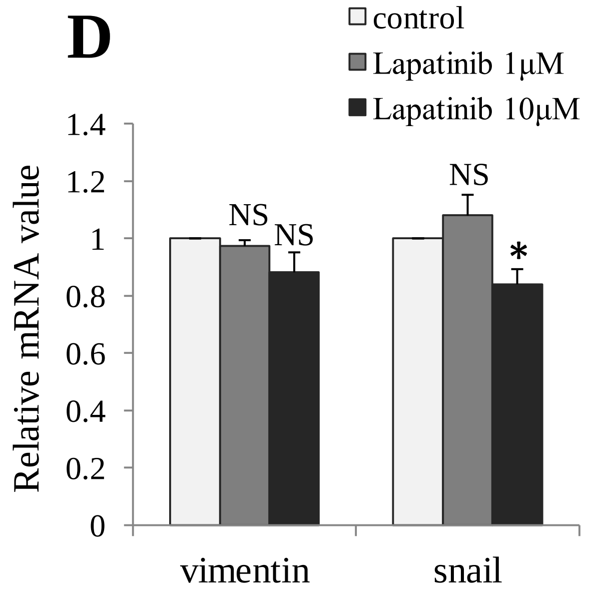

Figure 4S Effects of lapatinib and trastuzumab on the proliferation and EMT phenotype of 81B-Fb cells. (A) Growth inhibition of 81B-Fb cells by gefitinib, lapatinib and trastuzumab. (B) Western blot analysis of Akt, GSK-3β and snail expression in response to heregulin-β1 with or without lapatinib (LY294002) treatment. (C) Inhibition of motility of 81B-Fb cells by LY294002, lapatinib and trastuzumab treatment (12 h). (D) Effect of lapatinib treatment (24 h) on the vimentin and snail mRNA expression of 81B-Fb cells. Bars= SD, *$P<0.05$. NS=not significant.

Supplementary Table 1S

Sequences of primer-pairs for qRT-PCR used in this study

	Forward	Reverse	probe number
E-cadherin	cctggcctcagaagacagaa	ttgtctttgttggatttgatctg	# 44
vimentin	aaagtgtggctgccaagaac	agcctcagagaggtcagcaa	# 16
snail	gctgcaggactctaatccaga	atctccggaggtgggatg	# 11
twist	aaggcatcactatggactttctct	gccagtttgatcccagtatttt	# 6
slug	ttatgtttcctctaaaaacactgactg	tgcagtgagggcaagaaaa	# 7
EGF	aagaatgggggtcaaccagt	tgaagttggttgcattgacc	# 27
HB-EGF	tggggcttctcatgtttagg	catgcccaacttcactttctc	# 55
amphiregulin	cggagaatgcaaatatatagagcac	caccgaaatattcttgctgaca	# 38
EGFR	cgggacatagtcagcagtga	gggacagcttggatcacact	# 44
NRG1	cttgtggtcggcatcatgt	cagctttttccgctgtttct	# 49
NRG2	caaatatggcaacggcaga	ggcctcgcagacatactcc	# 39
GAPDH	agccacatcgctcagacac	gcccaatacgaccaaatcc	# 60
